# IP6 reduces colorectal cancer metastasis by mediating the interaction of gut microbiota with host genes

**DOI:** 10.3389/fnut.2022.979135

**Published:** 2022-09-02

**Authors:** Tong-Tong Lan, Yang Song, Xiao-Han Liu, Cui-Ping Liu, Hui-Chao Zhao, Yi-Sa Han, Chu-Hui Wang, Ning Yang, Zhen Xu, Meng Tao, Hui Li

**Affiliations:** ^1^Department of Nutrition and Food Hygiene, School of Public Health, College of Medicine, Qingdao University, Qingdao, China; ^2^Institute of STD and AIDS Prevention, Qingdao Municipal Center for Disease Control and Prevention, Qingdao, China; ^3^School of Nursing, College of Medicine, Qingdao University, Qingdao, China

**Keywords:** IP6, colorectal cancer, metastasis, gut microbiota, *Lactobacillus helveticus*, TNFRSF1B

## Abstract

Inositol hexaphosphate (IP6) is a phytochemical widely found in grains and legumes that plays an anti-cancer role. However, the mechanism underlying the inhibition of colorectal cancer metastasis by IP6 through host genes, gut microbiota, and their interactions remain elusive. In this study, 16S rRNA sequencing was used to study the effect of IP6 on gut microbiota in an orthotopic transplantation model of colorectal cancer mice. The transcriptome was used to study the changes of host genes in metastasis and the relationship with gut microbiota. The results showed that the gut microbiota composition of model mice was significantly different from that of normal mice. The beta diversity partly tended to return to the normal level after IP6 intervention. Especially, *Lactobacillus helveticus* and *Lactococcus lactis* were recovered after IP6-treated. Enrichment analysis showed that the enrichment score of the *Cytokine-Cytokine receptor interaction* signal pathway decreased after IP6 treatment compared to the model group. Further analysis of differentially expressed genes (DEGs) in this pathway showed that IP6 reduced the expression of the *Tnfrsf1b* gene related to the area of liver metastasis, and the *Tnfrsf1b* gene was negatively correlated with the relative abundance of *Lactobacillus helveticus*. Our results presented that host gene, microbiome and their interaction may serve as promising targets for the mechanism of IP6 intervention in colorectal cancer metastasis.

## Introduction

Colorectal cancer is the third most common cancer worldwide, with the second-largest mortality rate (1). The diagnosis and treatment of colorectal cancer have substantially improved in recent years, however, metastasis is still the leading cause of high mortality (2). It was reported that within 5 years of surgery, about a third of patients will develop metastasis, whereas 25 to 30% of patients with distant organ metastasis at the time of diagnosis (3, 4). Moreover, colorectal cancer patients with distant metastasis had a 5-year survival rate of only 10% (5). As a result, enhancing the prevention and treatment of colorectal cancer metastasis is important for the improvement of patients' survival rates.

Environmental and genetic factors play critical roles in the occurrence and development of colorectal cancer (6, 7), of which environmental factors contribute to around 80% of the risk (8). Dietary factor, among environmental factors, has a significant impact on the prevalence and progression of colorectal cancer (9). The western diet has been confirmed that linked to an increased risk of colorectal cancer (10). Conversely, consumption of whole grains is related to a decreased incidence of colorectal cancer (11). Such findings suggest that dietary interventions can successfully prevent or suppress colorectal cancer progression. Inositol hexaphosphate, commonly known as IP6, is a kind of phytochemical found in practically all plants and mammalian cells, particularly in wheat and bean. Wheat bran and flaxseed are the most abundant sources of IP6 (0.4 % to 6.4 %) (12). IP6 has been shown in studies to enhance Natural Killer cell (NK cell) activity, increase Tumor Necrosis Factor (*TNF-*α) expression, inhibit proliferation and induce apoptosis of colon cancer cells, which has shown an excellent anti-tumor activity (13, 14). According to epidemiological studies, IP6-rich diets (grains and legumes) are linked to a lower risk of colon cancer (15). IP6 can also decrease the growth of colorectal cancer in rats generated by 1, 2-dimethylhydrazine, enhance the expression of *E-cadherin*, whereas decrease the level of *TGF-*β to suppress intestinal mucosal barrier injury and inflammatory response (16). Researchers also find that IP6-riched diets could potentially affect tumor tissue that is outside of the gastrointestinal tract (17). IP6 can reduce the weight of liver tumors and the release of tumor marker fetoprotein (AFP) in HepG2 cells (Human hepatocellular carcinomas cells) subcutaneously implanted in nude mice (18). In addition, IP6 has also been demonstrated to suppress the metastasis of cancer. Intraperitoneal injection of IP6 can improve the survival rate of fibrosarcoma (FSA-1) mice model, with tumor size reduction, and slower lung metastasis progression (19). Thus, we hypothesize that IP6 can inhibit colorectal cancer spread to the liver. According to our prior research, IP6 inhibits liver metastasis in the mice model of colorectal cancer orthotopic transplantation via regulating gene expression, however, the potential mechanism has not been fully investigated (20).

Gut microbiota was illustrated related to the incidence and progression of colorectal cancer in a large number of studies in recent years (21–24). Researchers discovered that gut microbiota balance was altered in colon cancer patients, with substantial variations in composition and proportion when compared to the healthy people (25). Furthermore, the gut microbiota composition varies greatly depending on the stage of cancer (26). The gut microbiota of patients with colorectal cancer postoperative tumors is similar to that of cancer patients, while that of postoperative patients without tumors is similar to that of healthy people, which demonstrating that gut microbiota is related to colorectal cancer recurred after resection (27).

There are numerous facets to the mechanism of gut microbiota affecting colorectal cancer. Among them, pathogens can promote the progression of colorectal cancer. For example, *Enterotoxigenic Bacteroides fragilis* primarily by producing fragile toxins, which activate the β*-catenin* signaling pathway and *NF-*κ*B*, causing tumor cell excessive proliferation and inflammation, and thus promoting the progression of colorectal cancer (28, 29). In addition, the increase of beneficial bacteria in the gut can inhibit tumor development by increasing immune response and maintaining the integrity of intestinal mucosal barrier (30–32). For instance, *Lactobacillus casei*, a species of *Lactobacillus*, can boost the expression of *ZO-1* and prevent *TNF-*α and *INF-*γ-induced damage to the intestinal epithelial barrier function (33). Furthermore, recent animal studies demonstrate that gut microbiota dysregulation might modify the epigenetic transcriptome of host colorectal cancer epithelial cells, resulting in the change of gene expression and the metastasis of colorectal cancer epithelial cells (34). These findings illustrated that the changes of gut microbiota may link to host genes alterations in colorectal cancer metastasis.

The changes in gut microbiota structure and function can be modulated by short-term dietary treatments (35). According to epidemiological studies, diet might play a pivotal role in the progression of colorectal cancer through gut microbiota (36). Research has shown that undigested or partially digested IP6 can accumulate in the gastrointestinal system of animals as a dietary fiber supplement, affecting gut microbiota (37). Dietary IP6 supplementation has been demonstrated to enhance the amount of *Lactobacillus* in rat feces and promote the expression of *Mucin* in the intestinal, consequently protecting the intestinal mucosal barrier (37). Probiotics such as *Bifidobacterium* and *Lactobacillus* carry the gene-encoded phytase (IP6 hydrolytic enzyme) that creates active phytase can induce apoptosis of human colon cancer cells (HCT116) (38). It is suggest that IP6 may increase the growth of intestinal beneficial bacteria and with ability to ameliorate tumor proliferation. However, it still remains elusive whether IP6 impacts the gut microbiota of colorectal cancer with metastasis or not.

The purpose of present study was hypothesized that IP6 intervention might have protective effects of against colorectal cancer metastasis by regulating gut microbiota, gene expression and their interaction. The possible effect of IP6 on metastasis of colorectal cancer was measured by 16sr RNA sequence and transcriptome sequencing in orthotopic transplantation model. Our research may offer a new strategy for IP6 in the prevention of colorectal cancer metastasis.

## Materials and methods

### Cell line

The CT-26 mouse colorectal carcinoma cell line, provided by Shanghai Cell Bank (Chinese Academy of Sciences), was cultivated under conventional cell culture conditions (RPMI 1640 medium containing 10% serum, at 37 °C, 5% CO_2_). The lentiviral vector with Green fluorescent proteins (GFP) and luciferase (LUC)-expressing reporters were transfected into CT-26 cells (39). Puromycin treatment for 2 weeks selected cells with stable and high luciferase reporter expression, named CT-26-LUC.

### Animals, treatments and sample collection

**Figure 1:**
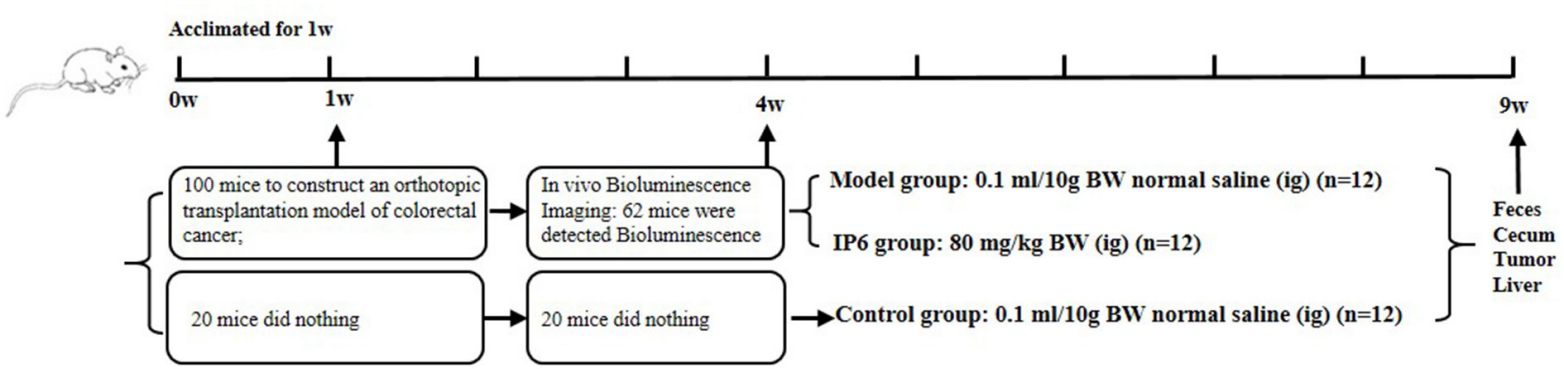
Study design showing model construction, *in vivo* Bioluminescence imaging, grouping, and intervention.

Six-week-old BALB/c male mice had ad libitum access to AIN93M standard feed (protein: 15%, fat: 9%, carbohydrate: 76%) and purified water and placed in the appropriate habitat (environment of specific pathogen-free (SPF), 12 h light/dark cycle; relative humidity around 52 ± 8%; temperature around 24 ± 2 °C). After 1 week of adaptive feeding, 100 of 120 mice were randomly selected to establish a mice model of orthotopic transplantation colorectal cancer with metastasis. Before the operation, mice were anesthetized with 0.1% pentobarbital sodium (80 mg/kg of body weight). The prepared CT-26-LUC cell suspensions (1 × 10^6^; 0.2 mL) were injected under the serosa of the cecum. The specific procedures refer to in reference (20). Three weeks after the operation, a total of 62 mice successfully detected bioluminescence using the PerkinElmer IVIS Spectrum Imaging System. 24 of 62 mice were randomly selected, with 12 mice given IP6 (80 mg/kg of weight) as the experimental IP6 group, and 12 mice given equal normal saline as the Model group (positive control) by gavage for 6 weeks. Another 12 mice were selected from the 20 mice that did not undergo the operation described above as the Control group (negative control) was treated with a volume of normal saline equal. The animals were monitored for tumor progression and liver metastasis 12 h after the last time of treatment using PerkinElmer IVIS Spectrum Imaging System and evaluated that progression using ImageJ software (Figure 1). The size of the liver metastatic area was reflected by the total number of photons in the luminescent region of mice. The animal study was reviewed and approved by the Medical Ethics Committee of Qingdao University, China (QYFYWZLL25667).

The feces were collected and stored at −80 °C for preservation. After that, Cervical dislocation was used to kill mice. The liver, cecum, cecum tumors and colon were dissected and weighed. The number of liver metastases was recorded. Then, such tissues were immediately submerged in liquid nitrogen and transferred to −80 °C.

### Microbiota analysis

The DNA extraction was performed using the CTAB (Hexadecyl trimethyl ammonium Bromide) method, and DNA concentration and purity were monitored on agarose gels. 16S rRNA genes of the V3-V4 region were amplified by PCR (98 °C for 1 min, followed by 30 cycles at 98 °C for 10 s, 50 °C for 30 s, 72 °C for 30 s, and finally 72 °C for 5 min) using optimized primers (338F, 5'-ACTCCTACGGGAG-GCAGCAG-3'; 806R, 5'-GGACTACHVGGGTWTCTAAT-3'). Following the manufacturer's instructions, sequencing libraries were created using the TruSeq® DNA PCR-Free Sample Preparation Kit (Illumina, USA). The library quality was assessed using the Qubit@ 2.0 Fluorometer (Thermo Scientific) and Agilent Bioanalyzer 2,100 system. The library was sequenced utilizing an Illumina NovaSeq platform and 250 bp paired-end reads were generated. Paired-end reads were merged utilizing FLASH (fast length adjustment of short reads) (Version V1.2.7), a very fast and accurate analysis tool (40). The splicing sequences were called raw tags. According to the QIIME (Version 1.9.1) (41), high-quality clean tags were obtained through quality filtering on the raw tags. Then the chimera sequences were eliminated after the tags were compared to the Silva database using the UCHIME algorithm (42). Finally, the Effective Tags were obtained. The Uparse algorithm (Uparse, Version 7.0.1001) was performed to assign sequences with ≥ 97% similarity to be same OTUs (43). The Silva 16S rRNA Database was used to annotate taxonomic information (44). Normalizing the OTUs abundance information was based on the sample with the least sequences.

### RNA-sequence analysis

The total RNA of cecal/ primary tumor tissue samples were prepared using TRIzol® Reagent (Invitrogen, USA) according to the manufacturer's instructions. Nanodrop2000 was used to detect the concentration and purity of the extracted RNA (45). Total RNA samples were converted to RNA-seq transcriptome libraries using Illumina Truseq^TM^ RNA Sample Kit. The library preparations were sequenced on an Illumina HiSeq (2 × 150 bp) platform. The RNA extraction, purification, reverse transcription, library construction, and sequencing were performed by Mei Ji Biotechnology Co., Ltd (Shanghai, China). For bioinformatics analysis, the expression level of transcripts was calculated by the FPKM (Fragments Per Kilobases per Million reads) method. Differential expression genes (DEGs) of raw counts were obtained by the DESeq2 package (Version1.10.1) in R (*p*-adjust < 0.05 & fold change > 1.5) (46). Functional enrichment analysis in KEGG pathways was performed to identify the enrichment of DEGs. The enrichment scores of pathways were calculated using Gene Set Enrichment Analysis (47).

### Statistical analysis

Statistical analysis was performed using GraphPad Prism 6 (GraphPad Software, San Diego, CA, USA). Data were represented as mean ± standard error (SE) of the mean. The one-way ANOVA was performed to compare the means for three groups with the Tukey post hoc test. The Kruskal-Wallis test was performed for nonparametric variables. Spearman correlation coefficient was used to represent the correlation between parameters. *P* < 0.05 was considered statistically significant.

## Results

### IP6 could alleviate metastasis in the orthotopic transplantation model of colorectal cancer mice

To investigate the effect of IP6 treatment on metastasis in the orthotopic transplantation model of colorectal cancer mice, we examined the liver metastasis rate, cecal tumor weights, liver weights, and the area of liver metastasis. The results showed that the percentage of death in the Model group (7/12) was significantly elevated compared with the Control group (0/12) (*P* < 0.05). While no statistical significance was founded between the IP6 group (3/12) and the Control group. The percentage of death in the IP6 group (3/12) was lower than that in the Model group (7/12) (*P* < 0.05). Additionally, the incidence of liver metastasis in the IP6 groups (3/12) was lower than that in the Model group (4/12), but the difference was not significant (*P* > 0.05). It's worth noting that the weights of cecal tumors and liver weights and the area of liver metastasis were found that the IP6 group was significantly lower than the Model group (*P* < 0.05) (Table 1).

**Table 1:** Effect of IP6 on the progression of colorectal cancer (mean ± SD).

**Group**	**Percentage**	**Liver metastatic**	**Primary tumor**	**Liver weight**	**Liver metastatic**
**name**	**of death (%)**	**rate (%)**	**weight (g)**	**(g)**	**area (%)**
Control	0.00	0.00	0.00	0.93 ± 0.10	0.00
Model	58.33^a^	33.33	5.90 ± 0.41	1.65 ± 0.18	47.25 ± 9.00
IP6	25.00^b^	25.00	4.27 ± 0.81^b^	1.37 ± 0.15^b^	21.25 ± 1.50^b^

a p < 0.05 compared to Control group.

b p < 0.05 compared to Model group.

### IP6-treated model mice showed apparent changes in gut microbial structure

We used 16S rRNA sequencing (V3-V4 region) of the feces to show how IP6 treatment affected the gut microbiota of the orthotopic transplantation model of colorectal cancer mice (*n* = 5 per group). From 15 fecal samples, a total of 908,807 effective sequences were recovered, with an average of 60,587 tags per sample (ranging from 48,621 to 69,624). Rarefaction curves and rank abundance curves flattened out suggesting that the majority of the microbiota diversity has been captured in all samples Supplementary Figure 1). While the three groups shared some overlapping regions and the IP6 mice had more of the same OTUs as control mice, we found no statistical differences in alpha diversity (observed-species, Shannon indexes, Chao1 index, and Simpson indexes) among Control, Model, and IP6 groups (*P* > 0.05) Supplementary Figure 2).

**Figure 2:**
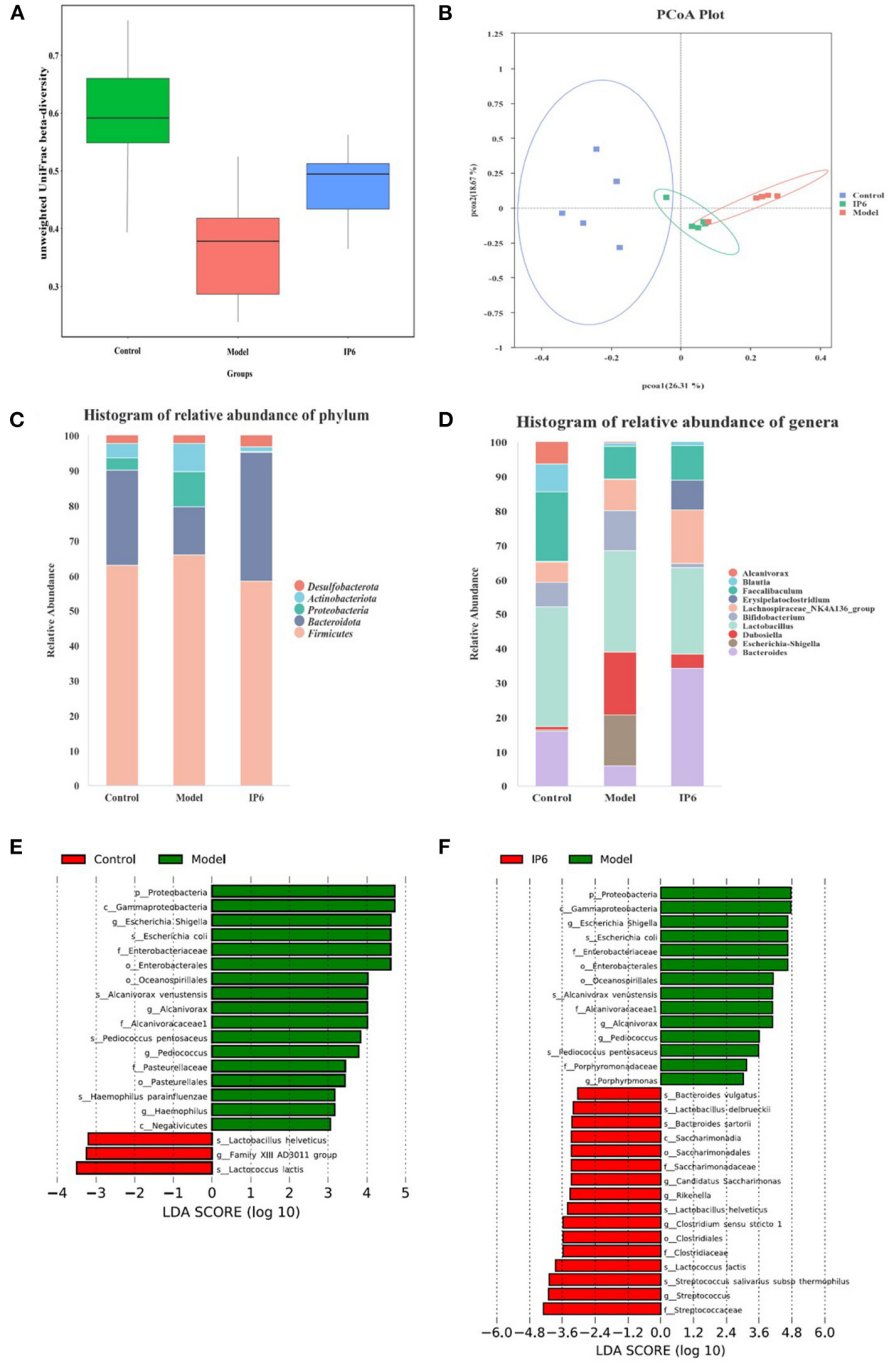
IP6 administration changes the composition of the gut microbiota in model mice. **(A)** Beta diversity of unweighted UniFrac distances analysis in gut microbiota. **(B)** The principal coordinates analysis (PCoA) of the unweighted UniFrac distances analysis. **(C)** Histogram of relative abundance of Top5 at the phylum level. **(D)** Histogram of relative abundance of Top10 at the genera level. **(E)** LDA scores of differentially abundant taxa between the Control and Model mice using the LEfSe method. **(F)** LDA scores of differentially abundant taxa between the Model and IP6 mice using the LEfSe method.

We found a significant difference in beta diversity of unweighted UniFrac of the fecal microbiome in the Model group compared to the Control group. IP6 treatments could reduce this difference (Model group VS IP6 group *p*-value = 0.021) (Figure 2A). The principal coordinates analysis (PCoA) of the unweighted UniFrac distances analysis showed that the samples in the Model group were separated from the Control group, while the IP6 group tended to cluster toward the Control group (Figure 2B). The phylum *Firmicutes* was dominant present in the gut microbiota from the three groups of mice, followed by *Bacteroidetes*. A higher abundance of phylum *Firmicutes, Proteobacteria*, and *Actinobacteriota*, and a lower abundance of *Bacteroidetes* were observed in the Model group, compared with the Control group, while supplementation of IP6 weakened alterations of *Bacteroidetes, Proteobacteria* and *Actinobacteriota* in Model group (Figure 2C). At the genus level, the Model group was different from the Control group in the relative abundance of the top 10 genera present in the gut microbiota, while the IP6 group was similar to the Control group (Figure 2D). Especially, the relative abundance of *Escherichia Shigella* in the model group was considerably greater than in the Control and IP6 groups (*P* = 0.047). In conclusion, IP6 could alter microbial community composition in mice to make them more resemble healthy mice.

Following that, LEfSe analysis of the fecal microbiota composition in the three groups was performed to further identify changes in phylum, genus, and species taxa after the IP6 intervention. Compared with the Model group, the Control group had lower abundances of *Escherichia Shigella, Alcanivorax, Pediococcus, Haemophilus, Escherichia coli, Alcanivorax venustensis, Pediococcus pentosaceus, Haemophilus parainfluenzaer* and higher abundance of *Lactobacillus helveticus, Family XIII AD3011 group*, and *Lactococcus lactis* (*P* < 0.05, LDA > 3, Figure 2E). Moreover, *Escherichia Shigella, Alcanivorax, Pediococcus, Porphyromonas, Escherichia coli, Alcanivorax venustensis*, and *Pediococcus pentosaceus* were enriched in the Model group, *Bacteroides vulgatus, Lactobacillus delbrueckii, Bacteroides sartorii, Candidatus Saccharimonas, Rikenella, Lactobacillus helveticus, Clostridium sensu stricto 1, Lactococcus lactis, Streptococcus salivarius subsp thermophiles*, and *Streptococcus* were increased by the IP6 treatment based on the model mice (*P* < 0.05, LDA > 3, Figure 2F). IP6 treatment might reduce the relative abundance of *Proteobacteria, Escherichia Shigella, Alcanivorax, Pediococcus, Escherichia coli, Alcanivorax venustensis*, and *Pediococcus pentosaceus* while increasing the relative abundance of *Lactobacillus helveticus* and *Lactococcus lactis* in model mice. From the results of the above LEfSe analyses, we obtained 1 phylum (*Proteobacteria*), 3 genera (*Escherichia Shigella, Alcanivorax*, and *Pediococcus*), and 5 species (*Escherichia coli, Pediococcus pentosaceus Alcanivorax venustensis, Lactobacillus helveticus, Lactococcus lactis*) for further processing.

### Cancer-related signaling pathways changes in the cecum of model mice was reversed by IP6

**Figure 3:**
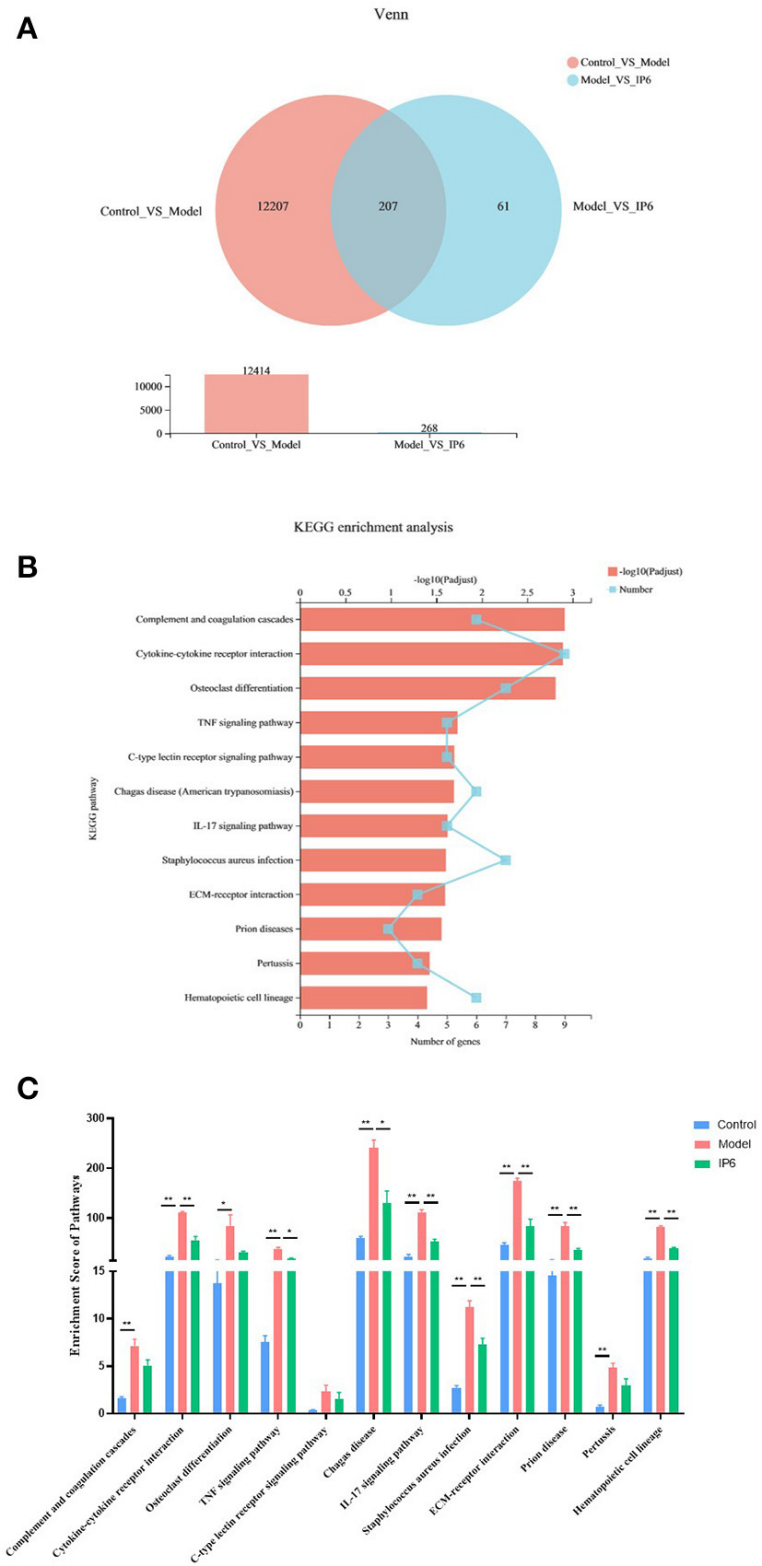
Partly signaling pathways changes in the cecum of Model mice be reversed by IP6-treated. **(A)** Venn Plot of the DEGs between the Control group vs. Model group and Model group vs IP6 group. **(B)** KEGG enrichment analysis line chart of 156 DEGs (Adjusted *P* < 0.05). **(C)** Enrichment scores of 12 KEGG signal pathways. Data were analyzed using one-way ANOVA followed by the Tukey *post hoc* test for C. *n* = 3 per group, *P < 0.05; **P < 0.01.

The transcriptome differences in the cecal of normal mice (Control group) and cecal tumor of model mice (Model and IP6 groups) were investigated using RNA-sequencing (*n* = 3 per group). There were 12,414 differentially expressed genes (DEGs) between the Model and Control groups (5,593 upregulated genes, 6,821 downregulated genes, Adjusted *P* < 0.05), and 268 DEGs between the Model and IP6 groups (144 upregulated genes, 124 downregulated genes, Adjusted *P* < 0.05), according to a comparative study of transcriptome profiles. There 207 DEGs were obtained from the intersection of differential genes between the Control group vs. Model group and Model group vs IP6 group (Figure 3A). It indicated that 207 of the DEGs between the Model and Control group could be regulated by IP6-treatment. Among them, 99 DEGs were downregulated and 57 DEGs were upregulated in the Model group when compared to the Control group, IP6 treatment could reverse the alterations of 156 DEGs by model.

To further investigate the biological signaling pathways implicated in the 156 DEGs, an enrichment analysis was done using the Kyoto Encyclopedila of Genes and Genomes (KEGG). These DEGs were discovered to be engaged in 190 signaling pathways, with 12 of them being highly enriched (Adjusted *P* < 0.05). These signaling pathways were primarily found in the Immune system, Signaling molecules and interactions, Signal transduction, Development and regeneration, and Infectious disease KEGG categories. Four out of 12 KEGG pathways were linked to the Immune system (Figure 3B).

Next, using gene set enrichment analysis (GSEA), we estimated the enrichment scores of KEGG pathways and analyzed the differences among the three groups. The results revealed that 8 candidate signaling pathways were identified for which the enrichment scores were increased due to model treatment but restored by the IP6 supplement, namely Cytokine-cytokine receptor interaction, TNF signaling pathway, Chagas disease, IL-17 signaling pathway, Staphylococcus aureus infection, ECM-receptor interaction, Prion disease, and Hematopoietic cell lineage (*p* < 0.05, Figure 3C).

### Correlations among the microbiota, signaling pathways, DEGs, and tumor-related parameters

**Figure 4:**
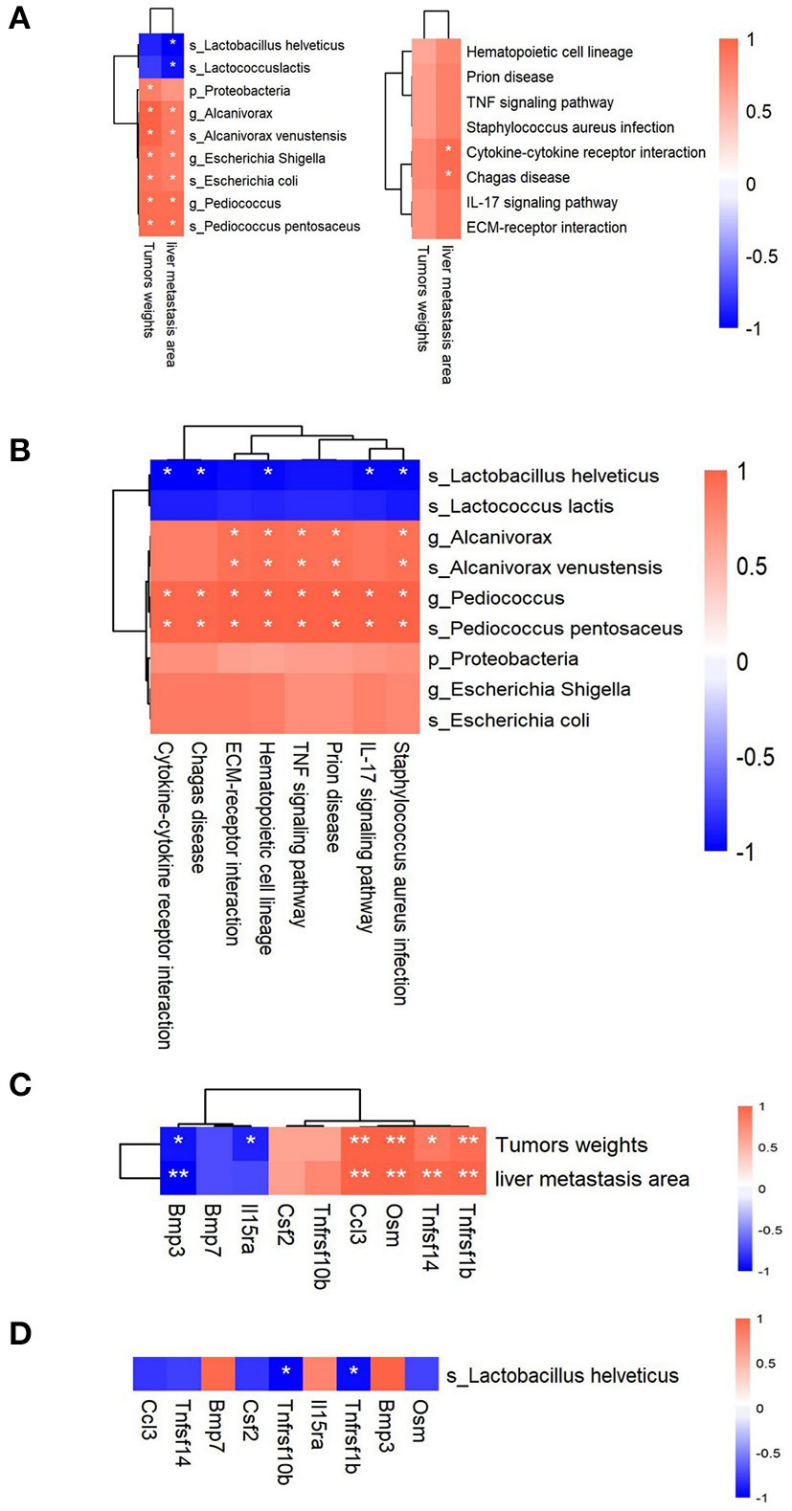
Correlations among the gut bacteria, signaling pathways, DEGs, and tumor-related parameters. **(A)** Heatmaps of the Spearman correlations between 2 tumor-related parameters and 9 microbiota taxa (Left) and 8 signaling pathways (Right). **(B)** Heatmap of the Spearman correlations between 9 microbiota taxa and 8 signaling pathways. **(C)** Heatmap of the Spearman correlations between 2 tumor-related parameters and 9 DEGs in the *Cytokine-Cytokine receptor interaction* signal pathway. **(D)** Heatmap of the Spearman correlations between *Lactobacillus helveticus* and 9 DEGs in the *Cytokine-Cytokine receptor interaction* signal pathway. *n* = 3 per group, *Adjusted *P* < 0.05; **Adjusted *P* < 0.01.

Here, 9 candidate microbiota taxa (2 taxa were enriched and 7 taxa were reduced in the IP6 group) and 8 KEGG signal pathways that can be restored by IP6 intervention could be obtained from the above analysis. To further analyze the association between the microbiota and KEGG signal pathway and metastasis indicators, Spearman correlation analysis was performed on the relationship among the weights of cecal tumors, the area of tumor metastasis, and 9 bacterial taxa and 8 KEGG pathways. The results showed that 1 phylum (*Proteobacteria*), 3 genera (*Escherichia Shigella, Alcanivorax, and Pediococcus*), and 3 species (*Escherichia coli, Pediococcus pentosaceus*, and *Alcanivorax venustensis*) were positively correlated with cecal tumor weights (Adjusted *P* < 0.05, Spearman rho > 0.7) (Figure 4A Left). Three genera (*Escherichia Shigella, Alcanivorax*, and *Pediococcus*) 5 species (*Escherichia coli, Pediococcus pentosaceus, Alcanivorax venustensis, Lactobacillus helveticus, and Lactococcus lactis*) were correlated with the area of tumors metastasis, in particular, *Lactobacillus helveticus* and *Lactococcus lactis* were significant negatively (Adjusted *P* < 0.05, Spearman rho > 0.7) (Figure 4A Left). Furthermore, cecal tumor weights were positively linked with cytokine-cytokine receptor interaction, ECM-receptor interaction, IL-17 signaling pathway, and Chagas disease (Spearman rho > 0.7). Only the Chagas disease and Cytokine-Cytokine receptor interaction signal pathways had significant positive relationships with tumor metastatic area in 8 positive candidate pathways (Adjusted *P* < 0.05, Spearman rho > 0.7) (Figure 4A Right).

Following that, we analyzed the correlations between 9 candidate microbiota taxa and 8 KEGG candidate pathways. *Alcanivorax* and *Alcanivorax venustensis* were shown to have significant positive correlations with 5 candidate pathways (TNF signaling pathway, Staphylococcus aureus infection, ECM-receptor interaction, Prion disease, and Hematopoietic cell lineage) (Adjusted *P* < 0.05, Spearman rho > 0.7). All 8 candidate pathways had significantly positive correlations with the *Pediococcus* and *Pediococcus pentosaceus* (Adjusted *P* < 0.05, Spearman rho > 0.7). Five candidate pathways (Cytokine-cytokine receptor interaction, Chagas disease, IL-17 signaling pathway, Staphylococcus aureus infection, and Hematopoietic cell lineage) exhibited strongly negative correlations with *Lactobacillus helveticus* (Adjusted *P* < 0.05, Spearman rho > 0.7) (Figure 4B).

Cytokine-Cytokine receptor interaction, in particular, showed a significant positive correlation with tumor metastasis areas and a significant negative correlation with *Lactobacillus helveticus*. We further analyzed the connections between the 9 DEGs in the *Cytokine-Cytokine receptor interaction* signal pathway and tumor metastasis indicators, as well as the connections between 9 DEGs and *Lactobacillus helveticus*. *Tnfrsf1b* was discovered to be associated with not only cecal tumor weights and liver metastatic area, but also *Lactobacillus helveticus* (Figures 4C,D). After IP6 intervention, *Lactobacillus helveticus* increased after IP6 intervention, which could be linked to *Tnfrsf1b* gene downregulation.

## Discussion

IP6 (Inositol hexaphosphate), a phytochemical found in natural grains and legumes, degrades mainly in the large intestine and plays an anticancer role by improving immunity, reducing cell proliferation, antioxidants, and inducing differentiation of malignant cells (13, 15). In the previous experimental results, IP6 showed an inhibitory effect on metastasis of colorectal cancer (20), but the mechanism of IP6 inhibiting colorectal cancer metastasis is not completely clear. In this study, a vivo model of orthotopic metastasis was established to simulate the whole process from primary to metastatic colorectal cancer. Our results suggest that colorectal cancer metastasis is associated with changes in the gut microbiota and host genes, and IP6 intervention can partially restore the changes in gut microbiota and signaling pathways caused by metastasis, thereby inhibiting the weights of the tumor in the cecum and the area of tumors metastasis, and improving survival rate.

Gut microbiota plays an important role in the progression of colorectal cancer (23, 26). In this study, the composition of the fecal microbiota of colorectal cancer metastasis model mice was significantly different from that of normal mice, the most important manifestation of which was the significant difference in beta diversity (Figure 2A). After IP6 intervention, the microbiota composition of model mice was more similar to that of normal mice (Figure 2B). The relative abundance of *Lactococcus lactis* and *Lactobacillus helveticus* reduced in the model mice were restored (Figures 2E,F). The relative abundance of *Lactococcus lactis* and *Lactobacillus helveticus* were significantly negatively correlated with the areas of tumor metastasis (Figure 4A). *Lactococcus lactis* and *Lactobacillus helveticus* belong to the genera *Lactococcus* and *Lactobacillus* respectively and are recognized as edible probiotics. *Lactobacillus* bacteria are common colonizers in the normal intestinal microecological structure of humans and animals and play important roles in host health (48). It has been reported that dietary IP6 supplementation could increase the abundance of *Lactobacillales* in rat fecal microbiota and promote the production of intestinal Mucin, thereby maintaining the intestinal mucosal barrier integrity (37). Among them, *Lactobacillus helveticus* was a kind of widely used *Lactobacillus*, which has a strong proteolytic ability (49). The research showed that the extracellular polysaccharide LHEPS-1 of *Lactobacillus helveticus MB2-1* could inhibit the proliferation of Caco-2 cells (50). *Lactobacillus helveticus* also have immunomodulatory effects. *Lactobacillus helveticus SBT2171* could increase the expression of regulatory T cells and decrease the production of pro-inflammatory cytokines (*IL-6* and *IL-1*β) in mice (51). *Lactobacillus helveticus NS8* has a good binding ability to human intestinal epithelial cells, which could promote the secretion of anti-inflammatory cytokine *IL-10*, and showed a considerable preventive effect on colitis in mice (52). In addition, *Lactobacillus helveticus* could also produce extracellular polysaccharides (EPS) with immunomodulatory activity in the fermentation process (53). As probiotic, *Lactobacillus helveticus* could also regulate the host flora and inhibit the proliferation of pathogenic bacteria (52). In our study, IP6 could promote the increase of *Lactobacillus helveticus*, and the strong proteolytic ability of *Lactobacillus helveticus* might help IP6 to decompose. Therefore, we hypothesized that *Lactobacillus helveticus* could not only play an anti-metastasis role but also help IP6 hydrolyzation to exert the anti-cancer effect. Another upregulated probiotic with IP6 intervention was *Lactococcus lactis*. It was generally recognized as a kind of harmless facultative anaerobic gram-positive lactic acid bacteria (LAB). *Lactococcus lactis* could produce metabolites with antibacterial activity (organic acids, bacteriocins, cyclic peptides, etc.) (54), and could inhibit the growth of harmful bacteria through competition (55). In our results, compared with healthy mice, the relative abundance of *Escherichia Shigella* and *Escherichia coli* in the Model group were increased, while the abundance of *Escherichia Shigella* and *Escherichia coli* in the IP6 group was significantly reduced (Figures 2E,F). *Escherichia Shigella* and *Escherichia coli*, both of which belong to the *Enterobacteriaceae*, had been identified as pathogenic bacteria closely associated with the occurrence and development of colorectal cancer. Recently, *Escherichia coli* C17 had been shown to stimulate the damage of the intestinal vascular barrier, thus allowing intestinal bacteria to spread across the barrier to the liver, and these bacteria create suitable “soil” for liver metastasis of colorectal cancer (56). Meanwhile, treated with *Escherichia coli*, tumors in situ were larger and had more metastases in mice (2). In this study, the reduction of IP6 on orthotopic tumor weights and liver metastasis areas was associated with a decrease in the relative abundance of *Escherichia coli* (Figure 4A). We have grounds to believe that the inhibitory effect of IP6 on pathogenic bacteria makes it play a better anti-metastasis effect.

Cytokines perform their biological functions by binding to corresponding cytokine receptors on the cell surface, which in turn initiate complex intracellular molecular interactions that ultimately lead to changes in cell gene transcription (cell signaling transduction). Selectively blocking the binding of cytokines, which were autocrine or paracrine from tumor cells to receptors and disrupting their self-regulated growth regulation mechanism is becoming an attractive research hotspot. Tumor necrosis factor -α (*TNF-*α) plays an important role in shaping the tumor microenvironment and coordinating tumor-microenvironment interaction (57). In this study, the Cytokine-Cytokine receptor interaction signaling pathway was positively correlated with the areas of tumor metastasis (Figure 4B), and the enrichment score (ES) of the Cytokine-Cytokine receptor interaction signaling pathway was decreased after IP6-treated (Figure 3C). *Tnfrsf1b*, equivalent to *TNFR2*, a gene in the Cytokine- Cytokine receptor interaction signaling pathway, had a significant positive correlation with the areas of tumor metastasis (Figure 4C). *Tnfrsf1b*, one of the receptors of tumor necrosis factor (*TNF*), had been identified as a survival factor, which could not only maintain cell survival and enhance proliferation but also participate in the adhesion and migration of tumor cells (58). It was reported that *Tnfrsf1b* could also enhance the phosphorylation of *STAT5*, which played a key role in immunosuppression (59, 60). At present, abnormal expression of *Tnfrsf1b* has been identified in at least 25 tumors, such as colon cancer, ovarian cancer, cutaneous T-cell lymphoma (CTCL), etc (61–64). Previous study found that targeting antagonism against *Tnfrsf1b* might be a potential therapeutic approach for tumors by simultaneously inhibiting *Treg* activity and inducing cancer cell death (65). Recently, several researchers have found that *Tnfrsf1b* played a key role in colonic liver metastasis. In male mice with *Tnfrsf1b-*deficient, the incidence of liver metastasis was significantly reduced, and *Tnfrsf1b* was considered to be a key regulator of the immunosuppressive microenvironment of colonic liver metastasis (66). Some population studies have also shown that *Tnfrsf1b* increased with the increase of the adenoma-carcinoma sequence, and this change was correlated with tumor-associated microorganisms (67). Therefore, inhibition of *Tnfrsf1b* might be used as a strategy to prevent the progression of colorectal cancer to the liver, while IP6 might be used as a reliable way to inhibit the expression of *Tnfrsf1b*, thus achieving the effect of inhibiting tumor metastasis. In addition, our study also found a negative correlation between *Tnfrsf1b* and *Lactobacillus helveticus* (Figure 4D). Studies had shown that the addition of *Lactobacillus helveticus* or mixed probiotics containing *Lactobacillus helveticus* could inhibit the production of *TNF-*α, thus playing a role in inflammation inhibition (68, 69). Unfortunately, the relationship between *Lactobacillus helveticus* and *Tnfrsf1b* had not been fully investigated yet, which deserve to be studied further.

In conclusion, IP6 showed potential prevention effects on metastasis of colorectal cancer in mice model. This might be due to the influence of IP6 in fecal microbiota, including increased the relative abundance of *Lactobacillus helveticus*, and decreased the relative abundance of *Escherichia coli, Escherichia Shigella* and other harmful bacteria. Furthermore, IP6 inhibited the expression of the metastasis*-*related gene *Tnfrsf1b*, which has a negative correlation with the abundance of *Lactobacillus helveticus*. However, the specific interaction mode between probiotics and host genes still needs further study.

## Data availability statement

The datasets presented in this study can be found in online repositories. The names of the repository/repositories and accession number(s) can be found at: https://www.ncbi.nlm.nih.gov/, PRJNA851023; https://www.ncbi.nlm.nih.gov/, PRJNA852872.

## Ethics statement

The animal study was reviewed and approved by Medical Ethics Committee of Qingdao University.

## Author contributions

T-TL and YS contributed to conception, and design of the study. T-TL wrote the manuscript. YS performed the funding, and monitoring of the project. X-HL and C-PL organized the database. YS, C-PL, and H-CZ contributed to revising the manuscript. T-TL, Y-SH, C-HW, and NY performed the statistical analysis. ZX, MT, and HL contributed to interpretation of data. All authors contributed to the article and approved the submitted version.

## Funding

This study was supported by the National Natural Science Foundation of China (81973033), and the Natural Science Foundation of Shandong Province (ZR2020MG057).

## Conflict of interest

The authors declare that the research was conducted in the absence of any commercial or financial relationships that could be construed as a potential conflict of interest.

## Publisher's note

All claims expressed in this article are solely those of the authors and do not necessarily represent those of their affiliated organizations, or those of the publisher, the editors and the reviewers. Any product that may be evaluated in this article, or claim that may be made by its manufacturer, is not guaranteed or endorsed by the publisher.
