# Supplementary material for: IP6 reduces colorectal cancer metastasis by mediating the interaction of gut microbiota with host genes
Source: Front Nutr. 2022 Sep 2;9:979135. doi: 10.3389/fnut.2022.979135 (PMC9479145; doi:10.3389/fnut.2022.979135)
Supplement: Supplementary file 1 [file Presentation_1.pdf]

Supplementary Material

1 Supplementary Figures

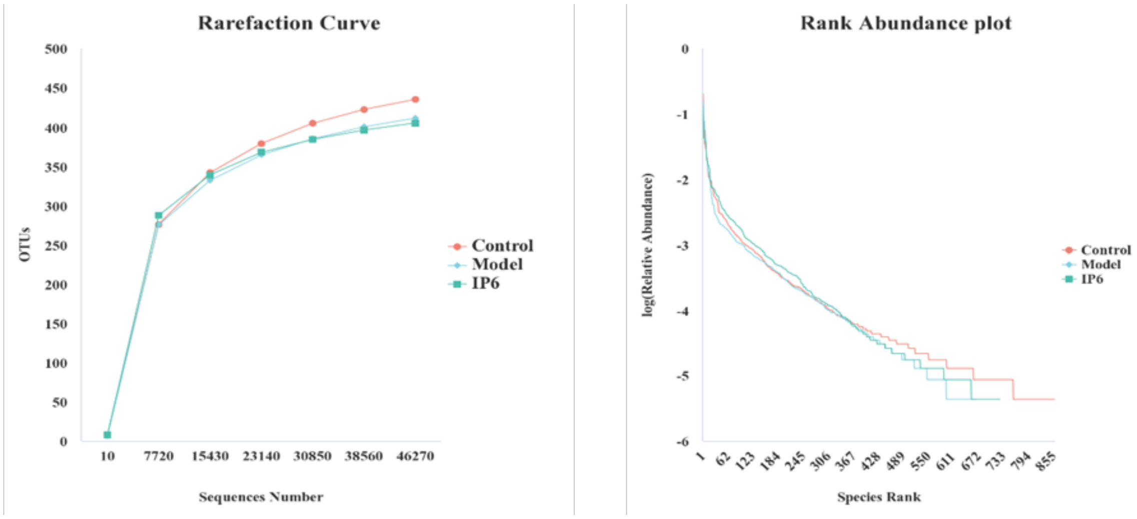

Supplementary Figure 1. Rarefaction curves and rank abundance in the three groups. Curves tend to be stable suggesting that the majority of the microbiota diversity has been captured in all samples.

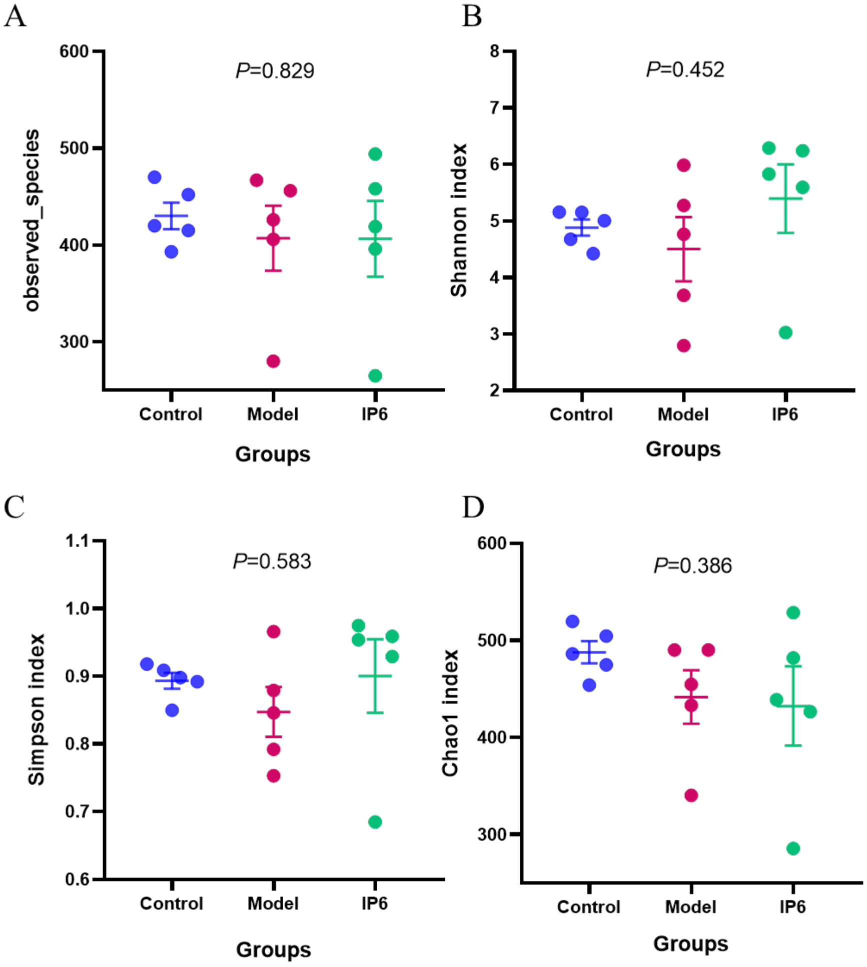

Supplementary Figure 2. alpha-diversity of observed-species (A), Shannon indexes (B), Chao1 indexes (C), and Simpson indexes (D) among Control, Model, and IP6 groups (n=5 per group).

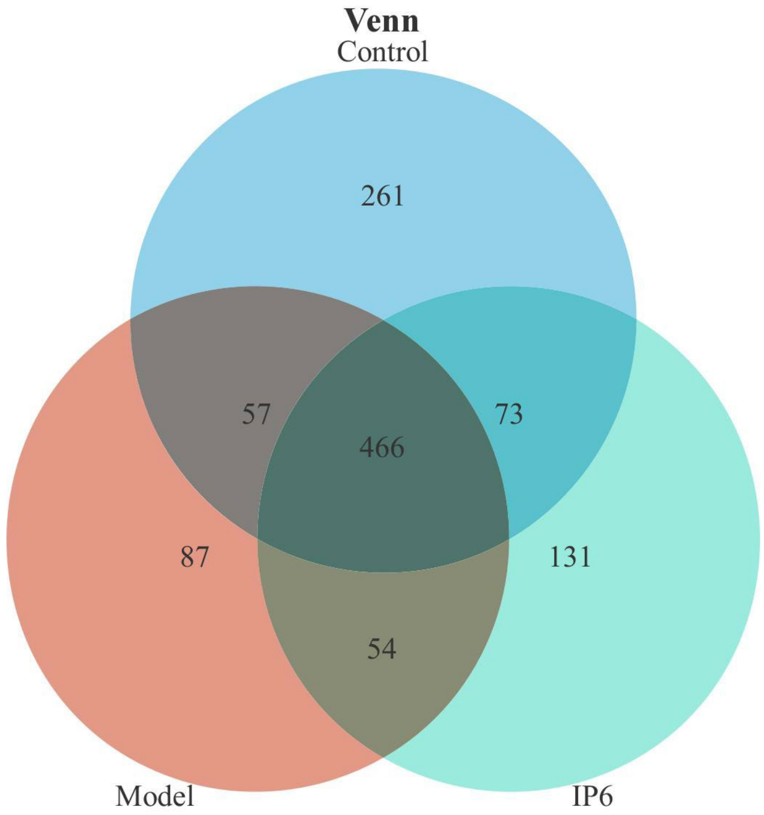

Supplementary Figure 3. Venn polt among Control, Model, and IP6 groups. The number above the graph represents the number of overlapping OTUs among the Control, Model, and IP6 groups (n=5 per group).

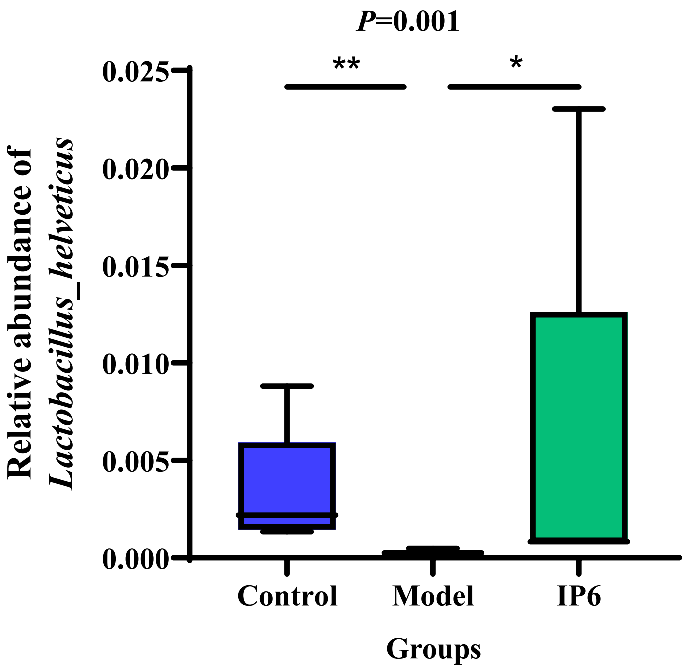

Supplementary Figure 4. Differences in the relative abundance of Lactobacillus_helveticus among Control, Model, and IP6 groups. The box plot with whiskers shows the median, maximum, and

minimum values. Kruskal-Wallis test was used for comparison among them with Dunn's multiple comparisons test. Control VS Model, **P =0.0052, IP6 VS Model, *P =0.0426, n= 5 per group.

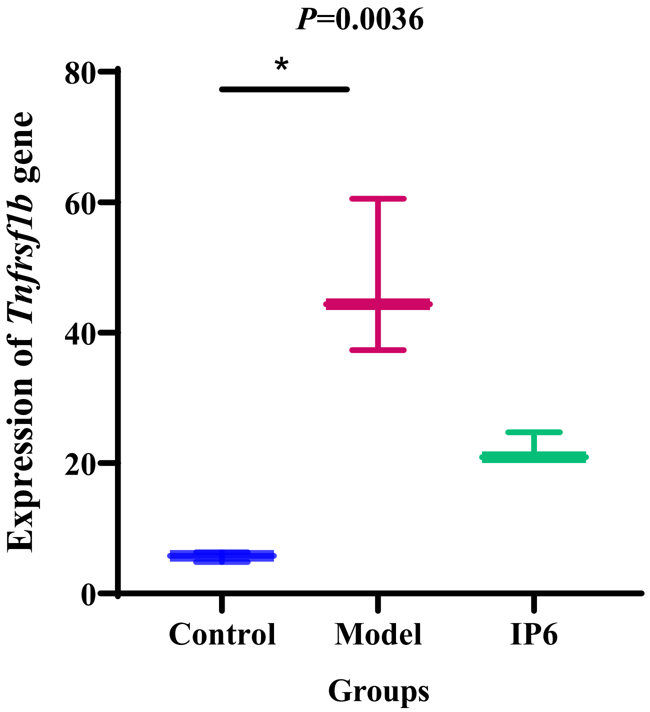

Supplementary Figure 5. Differences in expression of the Tnfrsf1b gene among Control, Model, and IP6 groups. The box plot with whiskers shows the median, maximum, and minimum values. Kruskal-Wallis test was used for comparison among them with Dunn's multiple comparisons test. Control VS Model, *P =0.0146, n= 3 per group.
